# Supplementary material for: The combination of CHK1 inhibitor with G-CSF overrides cytarabine resistance in human acute myeloid leukemia
Source: Nat Commun. 2017 Nov 22;8:1679. doi: 10.1038/s41467-017-01834-4 (PMC5698422; doi:10.1038/s41467-017-01834-4)
Supplement: Supplementary file 1 — Supplementary Information [file 41467_2017_1834_MOESM1_ESM.pdf]

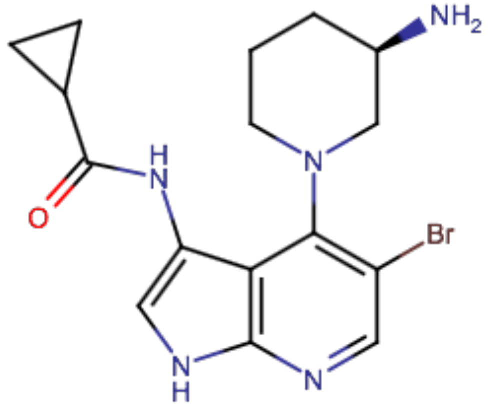

Supplementary Figure 1. Chemical structure of GDC-0575
Formula: $C_{16}H_{2O}BrN_5O$
Molecular Weight: 378.27
(*R*)-*N*-(4-(3-aminopiperidin-1-yl)-5-bromo-1*H*-pyrrolo[2,3-*b*]pyridin-3-yl) cyclopropanecarboxamide

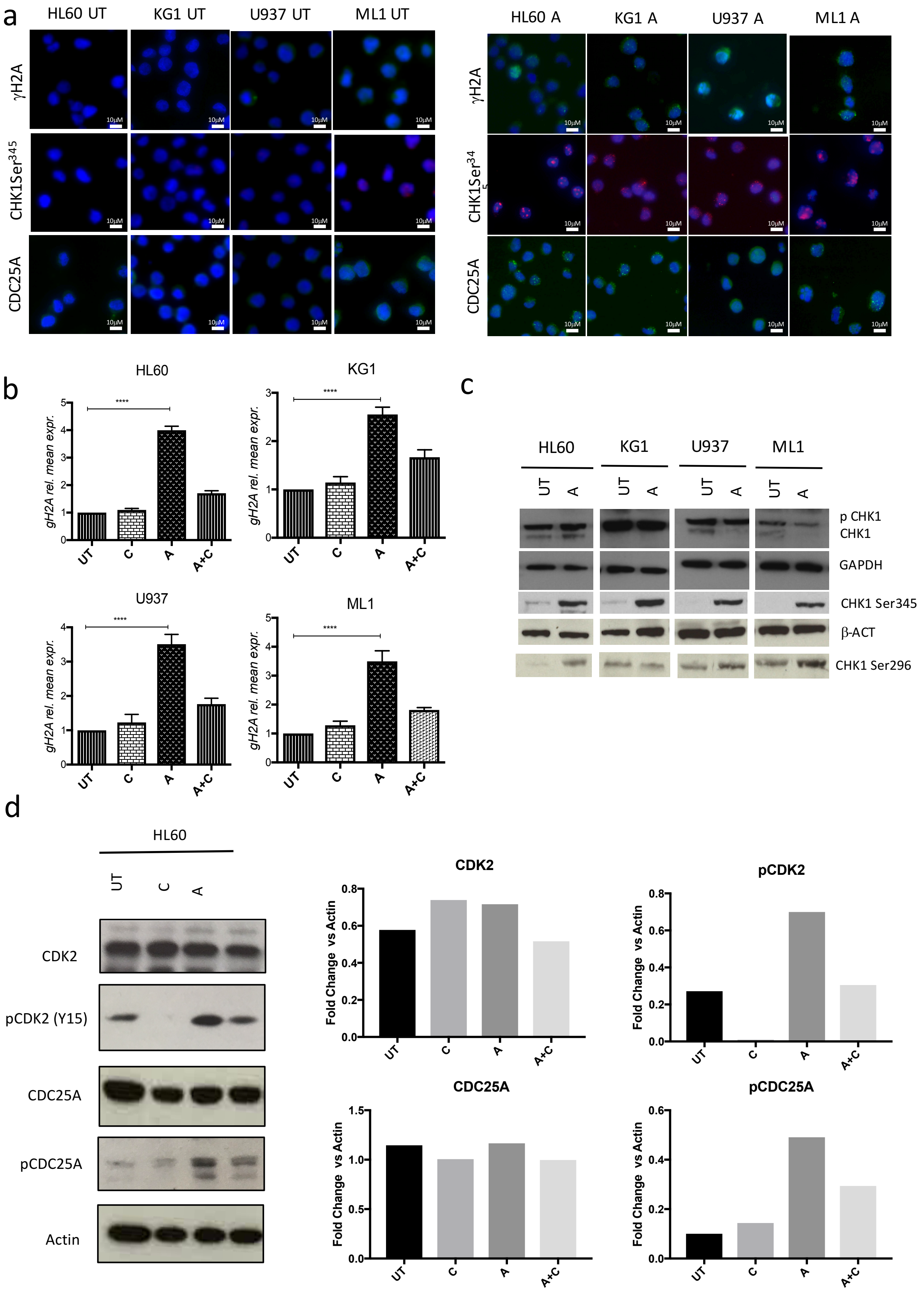

a
HL60 UT
KG1 UT
U937 UT
ML1 UT
HL60 A
KG1 A
U937 A
ML1 A
γH2A
CHK1Ser345
CDC25A
b
HL60
KG1
U937
ML1
gH2A rel. mean expr.
UT
C
A
A+C
c
p CHK1
CHK1
GAPDH
CHK1 Ser345
β-ACT
CHK1 Ser296
d
CDK2
pCDK2 (Y15)
CDC25A
pCDC25A
Actin
pCDK2
Fold Change vs Actin

Supplementary Figure 2. Ara.C induced DNA damag and activation of Chk.1. a) Immunofluorescence on AML cell lines untreated (left panel) or treated for 24h with AraC (right panel) showing activation of DNA damage-associated markers. **b)** Facs analysis showing the percentage of γH2A on the same AML cell lines as in a and under the described treatment conditions. **c)** Western blot showing both the global phosphorylation of Chk1 and its specific phosphorylation on the Ser345 and auto-phosphorylation Ser296 in AML cell lines upon 1d AraC treatment.

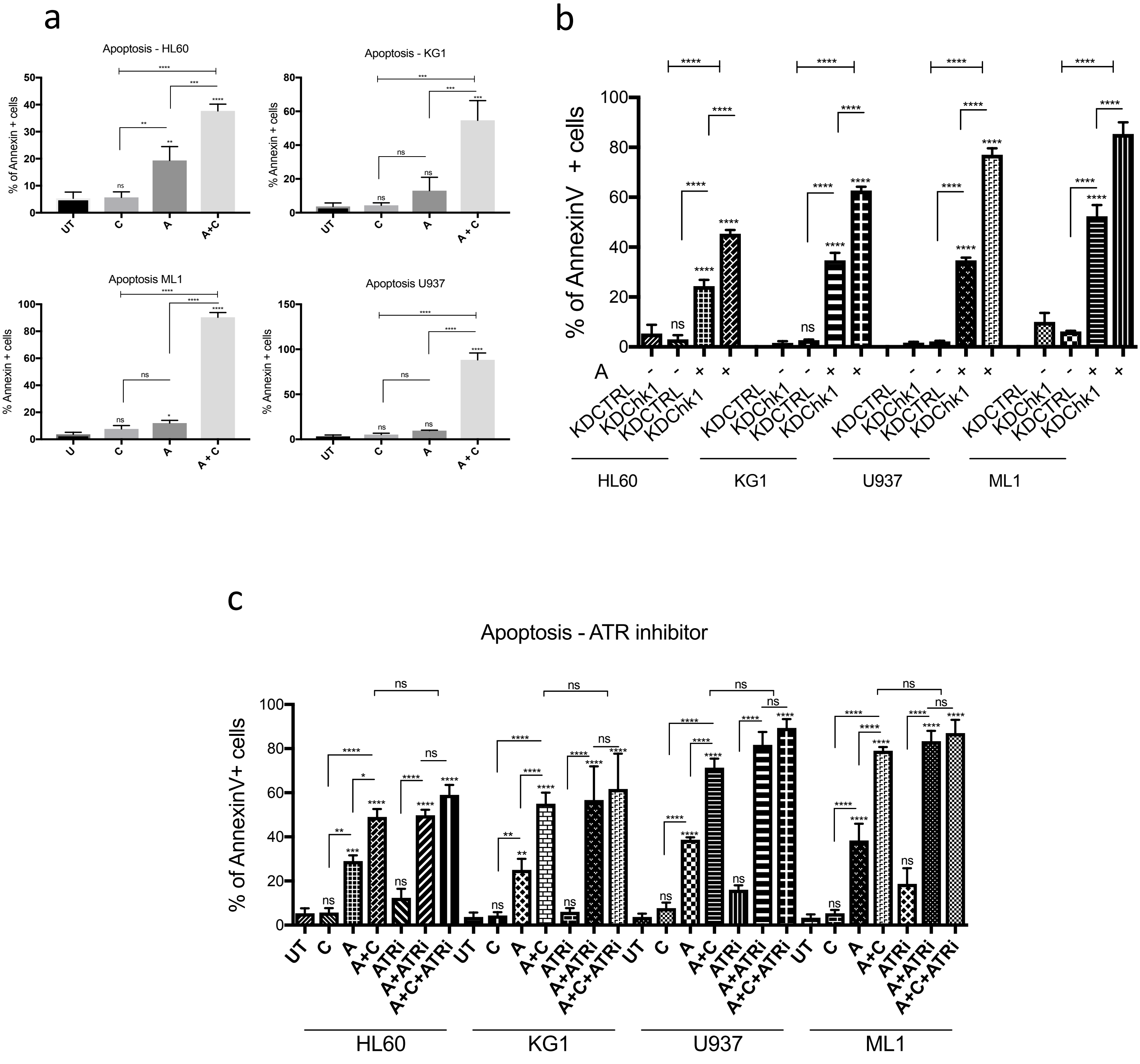

Supplementary Figure 3. CHK1inhibition via Chk.1 inh or KD of chk.1 or use of ATR inh enhance the cytotoxicity of AraC in different AML cell lines *in vitro* a) Percentage of apoptosis of four AML cell lines after 24hr of low dose Ara.C (100nM) ± Chk.1 inh; untreated (UT). UT n=3; Chk.1 (C) n=3, Ara.C (A) n=3 and A+C n=3. **b)** Percentage of apoptosis in four AML cell lines transfected with KDCTRL and KDChk1 after one-day treatment with AraC (A). n=3 for all samples. **c)** Percentage of apoptosis in four AML cell lines after 2 day treatment with A, AraC+CHK1i (A+C), ATR inhibitor (ATRi), A+ATRi, A+C+ATRi. n=3 for all samples.

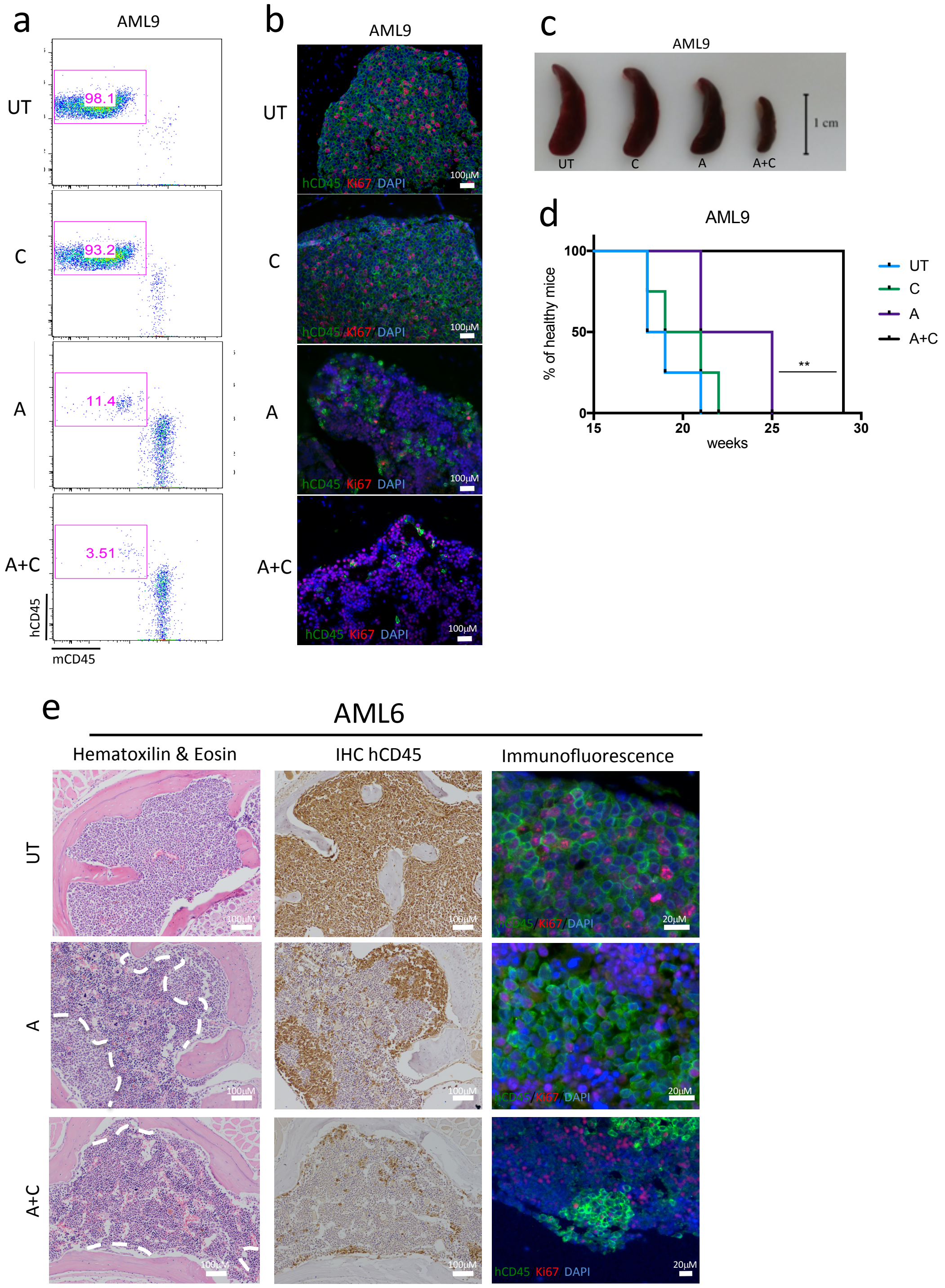

Supplemental Figure 4. CHK1i enhances the cytotoxicity of AraC in different primary AML *in vivo*. a) Percentage of human cells in the bone marrow of AML9-transplanted mice one week post-treatment. **b)** Immunofluorescence for Ki67 proliferation on mice's spine for the same patient and treatment conditions as in a. Scale bar is 100mM. **c)** Comparison of mouse spleens size for the same patient and treatment conditions as in a. **d)** Sickness curves monitoring AML9-injected mouse under different treatment conditions at various time points. (UT n=4; C n=4; A n=4; A+C n=4. Stat using Gahan-Breslow-Wilcoxon) **e)** Remaining cells in the bone marrow of AML6-transplanted mice one week post-treatment shown by H&E (left panel), CD45 IHC (middle panel) and IF (right panel; green cells are human CD45^{+}, red cells are Ki67^{+} and blue cells are the nuclei). Scale bar is indicated in the picture.

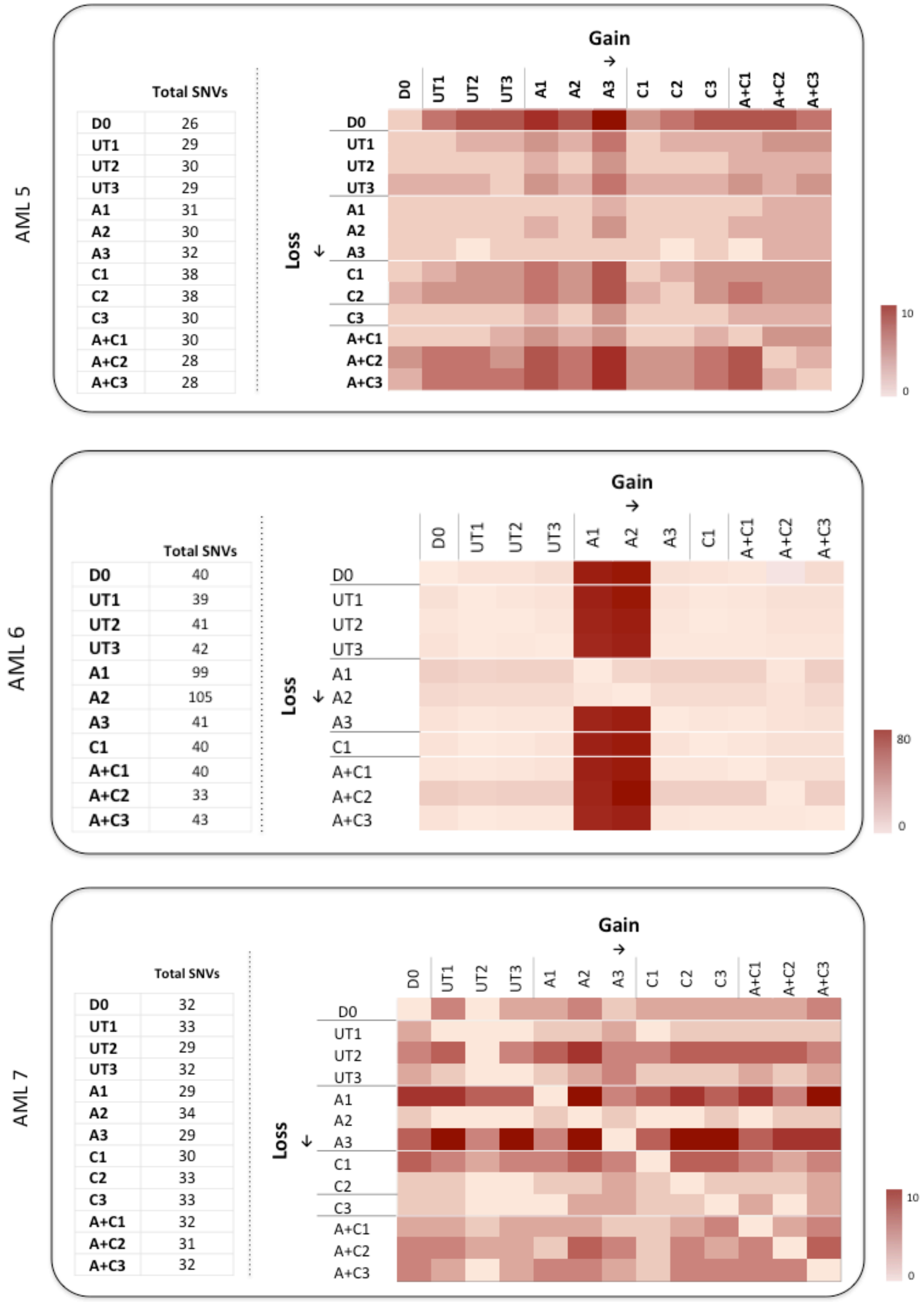

Supplemental Figure 5. CHK1i plus AraC treatment does not generate new mutations. Table (left) for each patient shows the total number of mutations (SNVs) detected in each condition. Heat-map (right) for each patient shows the changes in the mutation spectrum observed in each condition. Mutations present at D0 sample were compared to UT, A, C and A+C-mice.

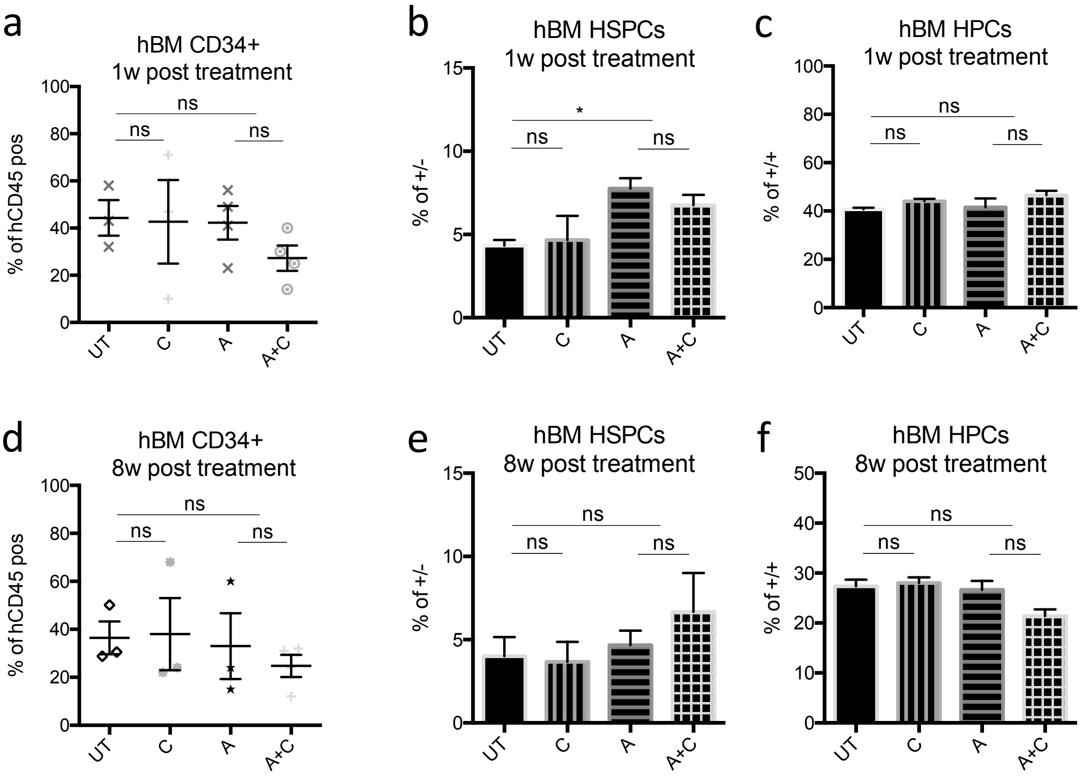

Supplementary Figure 6. CHK1i plus AraC treatment does not affect human hematopoiesis derived from bone marrow *in vivo*. a) Percentage of human cells in the BM aspirates of hBM-transplanted mice one week post-treatment. Each dot represents an individual mouse. UT n=3; C n=3; A n=4; A+C n=4. ns= not significant. Total number of mice= 14. **b)** Percentage of HSPCs (Lin$^-$CD34$^+$CD38$^-$) in the BM aspirates of hNM-transplanted mice one week post-treatment. The analysis was performed on the same mice as in a. **c)** Same as in b, but percentage of HPCs (Lin$^-$CD34$^+$CD38$^+$). **d)** Same as in a but 8 weeks post-treatment when mice were sacrificed for the analysis. **e)** Same as in B but 8 weeks post-treatment. **f)** Same as in c but 8 weeks post-treatment.

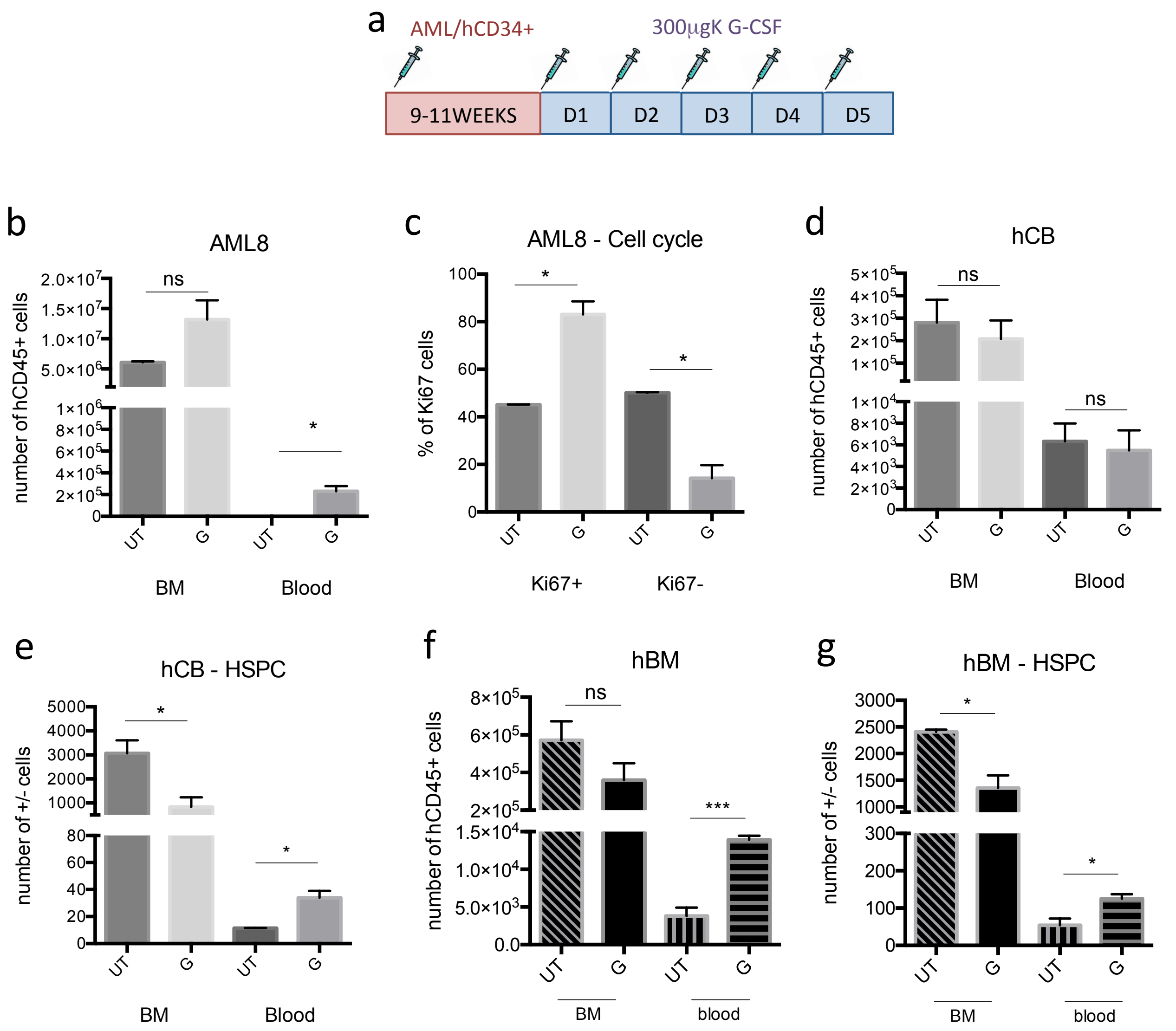

Supplementary Figure 7. G-CSF mobilizes both AML and normal hematopoietic cells *in vivo*. a) Treatment regimen for GCSF with AML or $hCD34^{+}$ cells, *in vivo*. **b)** Absolute number of $hCD45^{+}$ cells in AML8-transplanted mice after the treatment in BM and blood. UT n=3; G n=3. ns= not significant. **c)** Percentage of Ki67 positive and negative cells in the BM of AML8-transplanted mice after the treatment. UT n=3; G n=3. ns= not significant. **d)** Same as in b but mice were transplanted with hCB $CD34^{+}$ cells. UT n=4; G n=4. ns= not significant. **e)** Same as in d but looking at HSPC cells. **f)** Same as in b but mice were transplanted with hBM $CD34^{+}$ cells. UT n=4; G n=4. ns= not significant. **g)** Same as in f but looking at HSPC cells.

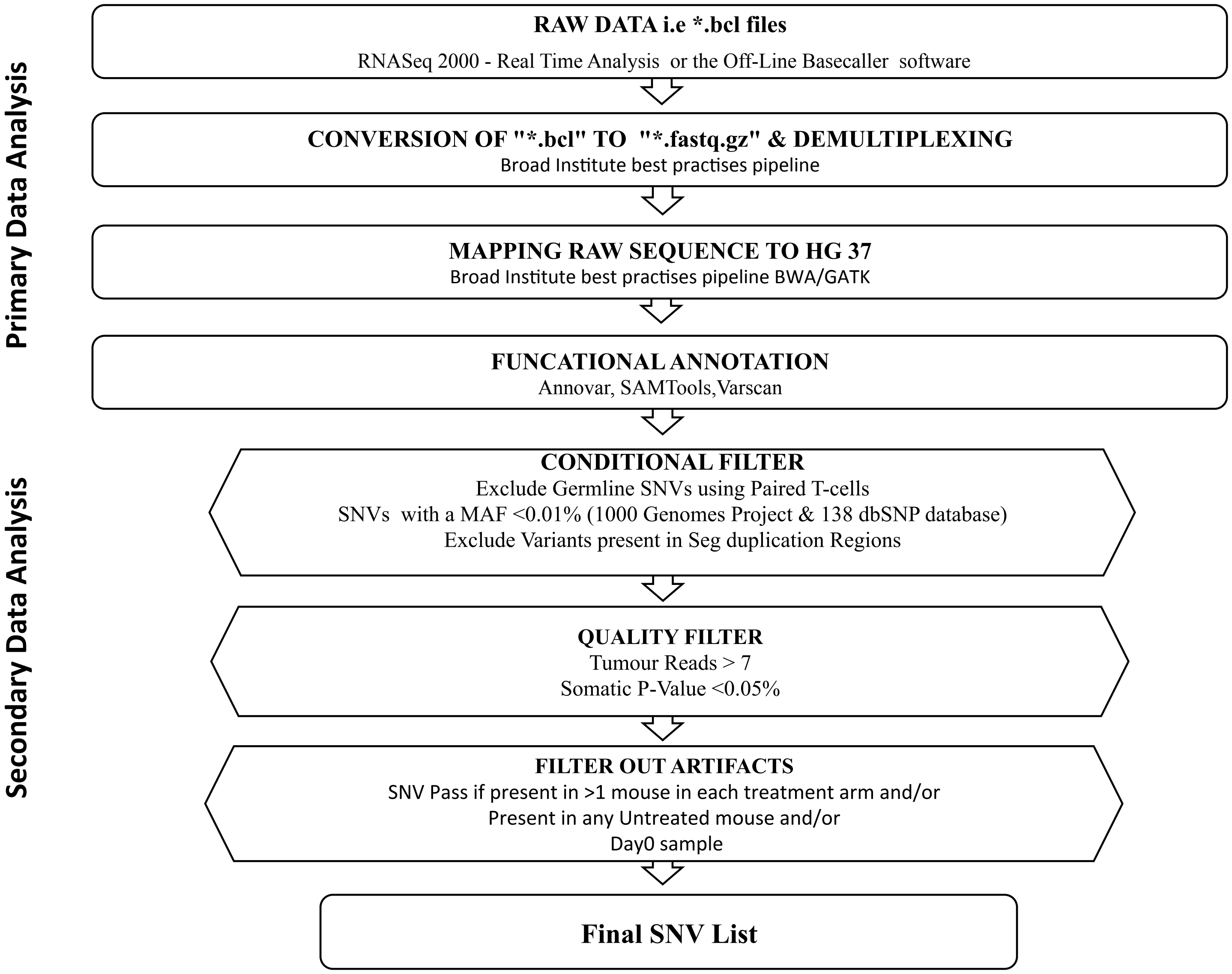

Supplementary Figure 8. Sequencing data analysis pipeline used for whole-exome sequencing data analysis. SNV-Single Nucleotide Variants

Supplementary Table 1. GDC-0575 Kinase Selectivity Profile (KinomeScan® at 100 nM)

% Residual Activity	n=460	Kinases Inhibited
< 1 %	n = 1	ChK1
5 - 20 %	n = 3	SLK, YSK4, MYLK
20 - 50%	n = 9	FLT3, GSG2, DAPK3, DMPK, MELK, CSNK2A2, JAK2, RPS6KA4, MARK3
50 -100%	n = 447	All others, including ChK2

ChK1 enzyme IC_{50} = 1.2 nM

*Additional IC_{50} determinations confirmed greater than 30-fold selectivity in potency for ChK1 inhibition over the off-target kinases (See Table Supplementary S2).

Supplementary Table 2. Description of AML patient samples features.

AML ID	FEATURE	CYTOGENETICS	CYTO RISK	NPM	FLT3	EXPERIMENT
AML1	Non Engrafter	Inv(16)	good	n.a.	n.a.	*in vitro*
AML2	Non Engrafter	t(9,6)	intermediate	n.a.	n.a.	*in vitro*
AML3	Engrafter	normal	intermediate	wt	ITD	*in vitro*
AML4	Engrafter	t(9,11)	intermediate	n.a.	n.a.	*in vitro*
AML5	Engrafter	complex	poor	wt	n.a.	*in vitro/in vivo*
AML6	Engrafter	normal	intermediate	mutant	ITD	*in vitro/in vivo*
AML7	Engrafter	normal	intermediate	mutant	ITD	*in vivo*
AML8	Engrafter	Del 7q+8+1	poor	wt	wt	*in vivo*
AML9	Engrafter	normal	intermediate	mutant	ITD	*in vivo*

Expt no	Gene	Ensembl_ID	Chr_position	Ref>Var	Amino Acid Change	Read 1	Read 2	Tumour Variant Frequency	Somatic P Value	cosmic70	SNP138_ 1000G	
AML5	LDLRAD3	ENSG00000179241	chr11_36248720	C>T	S131S	62	33	34.74%	1.27E-14	ID=COSM237653	rs142823958_0.0004	
AML5	STMN2	ENSG00000104435	chr8_80577757	G>A	UTR3	135	104	43.51%	6.79E-35	.	.	
AML5	FUZ	ENSG00000010361	chr19_50314725	C>T	splicing	58	73	55.73%	2.73E-22	.	.	
AML5	STK32B	ENSG00000152953	chr4_5128847	A>G	intronic	206	127	38.14%	1.77E-43	.	.	
AML5	WDR17	ENSG00000150627	chr4_177067106	A>G	intronic	28	26	48.15%	5.37E-07	.	.	
AML5	COL27A1	ENSG00000196739	chr9_116945255	C>T	intronic	20	8	28.57%	5.41E-04	.	.	
AML5	DSG2	ENSG00000046604	chr18_29099714	T>C	intronic	9	21	70%	1.37E-09	.	.	
AML5	SOGA1	ENSG00000149639	chr20_35425414	G>C	intronic	96	70	42.17%	4.33E-26	.	.	
AML5	RIMKLA	ENSG00000177181	chr1_42880383	A>T	N305I	236	60	20.27%	6.68E-15	.	.	
AML5	TTLL7	ENSG00000137941	chr1_84376926	A>C	Y570D	31	44	58.67%	3.88E-16	.	.	
AML5	NMI	ENSG00000123609	chr2_152132156	A>G	I159T	41	31	43.06%	9.55E-11	.	.	
AML5	FSIP2	ENSG00000188738	chr2_186654687	C>T	L1031F	73	38	34.23%	1.35E-17	.	.	
AML5	KPNA4	ENSG00000186432	chr3_160283015	C>G	G19A	104	50	32.47%	1.64E-16	.	.	
AML5	VCAN	ENSG00000038427	chr5_82785951	C>T	S35S	27	63	70%	1.07E-41	.	.	
AML5	STK31	ENSG00000196335	chr7_23825098	A>G	N694S	168	108	39.13%	6.41E-42	.	.	
AML5	TRPA1	ENSG00000104321	chr8_72958796	T>A	K671N	18	12	40%	1.09E-04	.	.	
AML5	NAA40	ENSG00000110583	chr11_63713403	A>G	N12S	43	38	46.91%	3.03E-13	.	.	
AML5	HYKK	ENSG00000188266	chr15_78825996	C>G	S369C	71	55	43.65%	8.81E-24	.	.	
AML5	XPO6	ENSG00000169180	chr16_28167651	C>G	G281R	142	91	39.06%	2.22E-35	.	.	
AML5	CNTNAP4	ENSG00000152910	chr16_76486419	T>G	F289L	77	62	44.60%	9.68E-26	.	.	
AML5	PLIN5	ENSG00000214456	chr19_4525739	C>T	R209H	135	98	42.06%	1.66E-40	.	.	
AML5	ZNF71	ENSG00000197951	chr19_57133272	G>A	R206H	237	95	28.61%	6.65E-26	.	.	
AML5	FBLN1	ENSG00000077942	chr22_45944540	C>T	R497C	35	36	50.70%	1.24E-16	.	.	
AML5	COL20A1	ENSG00000101203	chr20_61952223	C>T	intronic	7	5	41.67%	0.00555766	.	.	
AML5	ITIH3	ENSG00000162267	chr3_52833569	T>A	intronic	11	9	45%	7.93E-04	.	.	
AML5	TP53	ENSG00000141510	chr17_7577538	C>T	R116Q	20	70	77.78%	2.70E-33	ID=COSM99021	rs11540652	
AML7	QSOX1	ENSG00000116260	Chr1_180165613	C>T	P562L	145	142	49.48%	5.24E-13	.	.	
AML7	NLRP3	ENSG00000162711	Chr_1247587549	A>G	T268T	201	215	51.68%	4.43E-13	.	.	
AML7	APOB	ENSG00000084674	Chr2_21231407	C>T	G2778E	225	213	48.63%	1.28E-46	.	.	
AML7	GALNT5	ENSG00000136542	Chr2_158115938	C>G	P448P	81	81	50%	2.52E-10	.	.	
AML7	PLXNB1	ENSG00000164050	Chr3_48463201	G>A	S512F	71	61	46.21%	2.26E-05	.	.	
AML7	TRMT44	ENSG00000155275	Chr4_8477547	G>A	W698X	190	186	49.47%	0.00240843	.	.	
AML7	KLHL8	ENSG00000145332	Chr4_88104494	T>G	A88A	110	100	47.62%	1.44E-08	.	.	
AML7	SPEF2	ENSG00000152582	Chr5_35704757	C>G	Q834E	73	59	44.70%	2.21E-05	.	.	
AML7	CREB5	ENSG00000146592	Chr7_28610101	C>T	S104L	266	218	45.04%	6.03E-20	.	.	
AML7	KMT2E	ENSG00000005483	Chr7_104731706	G>A	S556N	64	45	41.28%	6.28E-06	.	.	
AML7	KIAA1549L	ENSG00000110427	Chr11_33566484	C>A	A685D	163	149	47.76%	1.93E-12	.		0.0002
AML7	ARNT2	ENSG00000172379	Chr15_80800561	G>A	M229I	94	74	44.05%	6.25E-11	.	.	
AML7	MYADM	ENSG00000179820	Chr19_54377434	G>A	A217A	287	300	51.11%	3.35E-26	.	.	
AML7	LAIR1	ENSG00000167613	Chr19_54868189	T>C	Y147C	70	75	51.72%	4.01E-05	.	.	
AML7	ZBBX	ENSG00000169064	Chr3_166966513	A>G	intronic	78	59	43.07%	6.38E-04	.	.	
AML7	ITGA1	ENSG00000213949	Chr5_52204889	T>C	intronic	80	77	49.04%	7.93E-05	.	.	
AML7	GLYATL3	ENSG00000203972	Chr6_49485109	G>A	intronic	104	120	53.57%	5.95E-21	.	.	
AML7	AIMP2	ENSG00000106305	Chr7_6054619	G>A	intronic	12	8	40%	0.04052952	.		0.0004
AML7	ZC3HC1	ENSG00000091732	Chr7_129685254	T>C	intronic	94	100	51.55%	2.23E-07	.	.	
AML7	CNGB3	ENSG00000170289	Chr8_87606558	C>T	intronic	20	16	44.44%	0.00486144	.	.	
AML7	VPS13B	ENSG00000132549	Chr8_100847343	A>G	intronic	39	36	48%	0.00455566	.	.	
AML7	BTAF1	ENSG00000095564	Chr10_93702171	T>G	intronic	67	18	21.18%	0.01817019	.	.	
AML7	NUP160	ENSG00000030066	Chr11_47834373	C>A	intronic	62	50	44.64%	1.84E-04	.	.	
AML7	BRMS1	ENSG00000174744	Chr11_66109761	G>C	intronic	46	73	61.34%	1.94E-10	.	.	
AML7	TMEM135	ENSG00000166575	Chr11_86782682	C>T	intronic	32	18	36%	5.81E-04	.	.	
AML7	SLC2A14	ENSG00000173262	Chr12_7970731	T>C	intronic	6	6	50%	0.00686499	.		0.001
AML7	NPAS3	ENSG00000151322	Chr14_34249952	C>A	intronic	133	75	36.06%	8.36E-10	.		0.0002
AML7	SPAG7	ENSG00000091640	Chr17_4863006	G>T	intronic	101	104	50.73%	9.70E-17	.	.	
AML7	CCDC40	ENSG00000141519	Chr17_78032910	A>G	intronic	22	20	47.62%	0.00662656	.	.	
AML7	IL1RAPL1	ENSG00000169306	ChrX_29935507	G>A	intronic	25	14	35.90%	0.04415273	.	.	
AML7	MAOB	ENSG00000069535	ChrX_43697074	G>A	intronic	20	17	45.95%	0.00161519	.		0.0013
AML7	NCOR1	ENSG00000141027	Chr17_16062188	C>T	splicing	32	39	54.93%	0.00151075	.	.	
AML6	EBF2	ENSG00000221818	chr8_25702020	G>A	UTR3	24	28	53.85%	8.56E-14	.	.	
AML6	SLAMF7	ENSG00000026751	chr1_160721107	C>T	intronic	54	48	47.06%	4.22E-19	.	.	
AML6	TBC1D1	ENSG00000065882	chr4_38037425	A>G	intronic	16	20	55.56%	1.87E-07	.	.	
AML6	SPEF2	ENSG00000152582	chr5_35790110	C>T	intronic	151	77	33.77%	1.29E-32	.	.	
AML6	KIF13B	ENSG00000197892	chr8_28967641	C>T	intronic	35	34	49.28%	7.62E-15	.	.	
AML6	HSPA12A	ENSG00000165868	chr10_118440546	G>A	intronic	35	20	36.36%	4.09E-06	.		0.0002
AML6	NOX4	ENSG00000086991	chr11_89224224	G>A	intronic	37	33	47.14%	3.67E-13	.	.	
AML6	FREM2	ENSG00000150893	chr13_39451239	T>C	intronic	76	97	56.07%	3.67E-44	.	.	
AML6	LDLRAD4	ENSG00000168675	chr18_13611030	G>A	intronic	21	20	48.78%	5.02E-10	.	.	
AML6	SYMPK	ENSG00000125755	chr19_46366108	G>A	intronic	27	29	51.79%	1.30E-12	.	.	
AML6	AP2A1	ENSG00000196961	chr19_50306467	G>A	intronic	73	55	42.97%	2.85E-20	.	.	
AML6	ATP6V1E1	ENSG00000131100	chr22_18082755	G>A	intronic	20	42	67.74%	1.26E-15	.	.	
AML6	SYN1	ENSG00000008056	chrX_47464548	G>A	intronic	34	16	32%	3.50E-05	.	.	
AML6	LRRTM4	ENSG00000176204	chr2_77746270	C>T	R243H	100	104	50.98%	5.56E-42	ID=COSM266868	.	
AML6	TET2	ENSG00000168769	chr4_106156729	C>T	R544X	117	114	49.35%	7.55E-44	ID=COSM41850	.	
AML6	CMYA5	ENSG00000164309	chr5_79034662	C>T	H3358H	89	75	45.73%	4.11E-25	.	.	
AML6	DNAH11	ENSG00000105877	chr7_21730446	C>T	N1996N	84	83	49.70%	2.12E-33	.	.	
AML6	SVEP1	ENSG00000165124	chr9_113242031	G>A	R791C	111	85	43.37%	1.68E-33	.	.	
AML6	AHNAK	ENSG00000124942	chr11_62294702	T>C	K2396R	68	37	35.24%	3.87E-17	.	.	
AML6	RYR3	ENSG00000198838	chr15_33842399	G>A	R285Q	60	28	31.82%	6.02E-09	.	.	
AML6	DCC	ENSG00000187323	chr18_50976873	C>T	P1078L	16	7	30.43%	1.50E-04	ID=COSM226365	.	
AML6	KIF16B	ENSG00000089177	chr20_16360726	G>A	R641C	60	69	53.49%	2.42E-25	ID=COSM1681548	.	
AML6	KLHL13	ENSG00000003096	chrX_117044028	C>T	R195Q	60	67	52.76%	2.68E-23	.	.	
AML6	PRKCB	ENSG00000166501	chr16_24043511	T>C	C115R	67	58	46.40%	4.57E-23	.	.	
AML6	CSE1L	ENSG00000124207	chr20_47675011	G>A	S4N	16	15	48.39%	1.03E-06	.	.	
AML6	CENPJ	ENSG00000151849	chr13_25485908	G>A	intronic	13	15	53.57%	7.04E-07	.	.	
AML6	ACTN2	ENSG00000077522	chr1_236914648	G>A	intronic	11	9	45%	6.15E-04	.	.	
AML6	UNC79	ENSG00000133958	chr14_94041513	G>A	S550N	73	72	49.66%	1.17E-30	.	.	
AML6	CELSR1	ENSG00000075275	chr22_46787632	C>T	D2016N	23	19	45.24%	1.76E-07	.		0.0004
AML6	AP2M1	ENSG00000161203	chr3_183895917	G>A	intronic	67	43	39.09%	5.72E-14	.	.	
AML6	HBS1L	ENSG00000112339	chr6_135317932	A>G	T274T	97	75	43.60%	1.40E-33	.	.	
AML6	GFRA2	ENSG00000168546	chr8_21608385	A>G	L37P	84	60	41.67%	1.61E-25	.	.	
AML6	SBF2	ENSG00000133812	chr11_10051353	T>G	N158H	30	34	53.12%	7.47E-18	.	.	
AML6	CYTH1	ENSG00000108669	chr17_76694387	A>C	F200V	38	21	35.59%	4.96E-10	.	.	
AML6	SHANK1	ENSG00000161681	chr19_51217442	C>T	G213R	23	22	48.89%	2.49E-08	.	.	
AML6	GPR101	ENSG00000165370	chrX_136113330	G>A	Y168Y	58	58	50%	4.96E-22	ID=COSM3231809	.	
AML6	YTHDF3	ENSG00000185728	chr8_64122434	C>A	UTR3	10	7	41.18%	0.01070425	.	.	
AML6	D2HGDH	ENSG00000180902	chr2_242694669	A>G	intronic	32	21	39.62%	5.53E-06	.	.	
AML6	PPP5D1	ENSG00000230510	chr19_46984436	A>G	UTR3	2	6	75%	0.0015083	.	.	
AML6	KCNN3	ENSG00000143603	chr1_154841416	C>A	intronic	10	9	47.37%	0.00130833	.	.	

Supplementary Table 3. Table showing the whole-exome Sequencing data for 3 Patient samples. Primary CD33+ cells from each patient were used for whole-exome sequencing. High-confidence mutations were selected following the primary data and secondary data analysis. WGA-Whole Genome Amplification.

		10 Reads	20 Reads	30 Reads	40 Reads	50 Reads	60 Reads	70 Reads	80 Reads	90 Reads	100 Reads
AML6_D0	Depth Coverage Level	98%	96%	94%	91%	87%	84%	80%	76%	72%	68%
AML6_T		98%	97%	95%	93%	91%	88%	86%	83%	80%	77%
AML6 UT1		98%	95%	92%	88%	83%	79%	74%	69%	65%	60%
AML6 UT2		98%	97%	95%	93%	90%	87%	84%	81%	78%	74%
AML6 UT3		98%	96%	94%	91%	87%	84%	80%	76%	73%	69%
AML6 A1		98%	97%	94%	92%	89%	85%	82%	78%	75%	71%
AML6 A2		98%	96%	94%	90%	87%	83%	79%	75%	71%	67%
AML6 A3		98%	97%	95%	93%	91%	88%	85%	82%	79%	75%
AML6 C1		98%	95%	92%	88%	83%	79%	74%	69%	65%	60%
AML6 A+C1		98%	97%	94%	91%	88%	85%	81%	77%	73%	69%
AML6 A+C2		98%	94%	91%	86%	81%	76%	70%	65%	60%	55%
AML6 A+C3		98%	95%	91%	86%	82%	76%	71%	66%	61%	56%
AML5_D0		98%	96%	92%	89%	84%	80%	75%	71%	66%	62%
AML5_T		98%	96%	94%	92%	89%	85%	82%	79%	75%	71%
AML5 UT1		98%	96%	92%	89%	85%	81%	76%	72%	68%	63%
AML5 UT2		99%	97%	96%	94%	91%	89%	86%	83%	80%	77%
AML5 UT3		98%	97%	94%	92%	89%	85%	82%	78%	75%	71%
AML5 A1		98%	96%	94%	91%	88%	84%	80%	77%	73%	69%
AML5 A2		98%	96%	94%	91%	88%	84%	80%	77%	73%	69%
AML5 A3		99%	97%	95%	93%	91%	88%	86%	83%	80%	77%
AML5 C1		98%	96%	93%	90%	87%	83%	79%	75%	71%	67%
AML5 C2		98%	96%	93%	90%	86%	82%	78%	74%	70%	66%
AML5 C3		98%	96%	94%	91%	88%	84%	80%	77%	73%	69%
AML5 A+C1		98%	95%	91%	87%	82%	77%	72%	67%	62%	57%
AML5 A+C2		98%	95%	90%	86%	81%	75%	70%	65%	60%	55%
AML5 A+C3		98%	94%	90%	86%	81%	76%	71%	66%	61%	57%
AML7_D0		99%	98%	96%	95%	93%	92%	90%	88%	86%	84%
AML7_T_wga		53%	39%	31%	25%	21%	18%	15%	13%	12%	10%
AML7 UT1		99%	98%	96%	95%	93%	91%	89%	87%	84%	82%
AML7 UT2		97%	92%	87%	80%	74%	67%	60%	54%	48%	42%
AML7 UT3		99%	98%	96%	94%	92%	90%	87%	84%	81%	78%
AML7 A1		96%	92%	86%	79%	72%	65%	58%	51%	45%	39%
AML7 A2		98%	97%	95%	92%	89%	87%	83%	80%	77%	73%
AML7 A3		97%	93%	87%	81%	75%	68%	62%	55%	49%	44%
AML7 C1		98%	95%	91%	87%	82%	77%	72%	67%	62%	57%
AML7 C2		97%	94%	90%	85%	80%	74%	68%	63%	57%	52%
AML7 C3		99%	97%	96%	94%	92%	90%	87%	84%	82%	79%
AML7 A+C1		97%	93%	88%	83%	76%	70%	63%	57%	51%	45%
AML7 A+C2		97%	94%	89%	84%	78%	72%	66%	61%	55%	50%
AML7 A+C3		98%	95%	89%	80%	71%	61%	52%	44%	36%	29%

Supplementary Table 4. Whole-exome Sequencing coverage data. Sequencing coverage across the exonic regions for primary patient CD33+ D0 cells, post-transplanted xenografted cells for all patients samples used in this study.

GROUP	SECONDARY ENGRAFTMENT OF hBM CELLS > 0.05%
UT	2/4
C	1/4
A	2/2
A+C	1/2
A+C+G	1/2

Supplementary Table 5. Secondary transplantation of hCD45$^+$ cells, pooled from hBM-transduced mice one week post-treatment. Mice were sacrificed at 16w. Mice engraftment with >0.05% human cells were considered positive. UT n=4; C n=4; A n=2; A+C n=2; A+C+G n=2.
